# *In vitro* comparison of methods for sampling copper-based antimicrobial surfaces

**DOI:** 10.1128/spectrum.02441-23

**Published:** 2023-10-17

**Authors:** T. C. Williams, T. Woznow, B. Velapatino, E. Asselin, D. Nakhaie, E. A. Bryce, M. Charles

**Affiliations:** 1 Division of Medical Microbiology and Infection Prevention, Vancouver Coastal Health, Vancouver, British Columbia, Canada; 2 Department of Materials Engineering, University of British Columbia, Vancouver, British Columbia, Canada; 3 Department of Pathology and Laboratory Medicine, University of British Columbia, Vancouver, British Columbia, Canada; Health Canada, Ottawa, Canada

**Keywords:** Cu surfaces, self-disinfecting surfaces, infection control, antibacterial measurement

## Abstract

For more than a decade, copper (Cu) and Cu alloy surfaces have been approved and registered as solid antimicrobials to reduce the environmental bioburden of potential pathogens. Bacterial collection and enumeration are important steps in assessing antimicrobial efficacy of these surfaces, yet comparisons of the different collection methods from Cu surfaces are scarce in the literature. This study compared Petrifilm (PF) aerobic count plates applied directly onto Cu surfaces and two indirect (cellulose sponges and Quick Swab) collection methods to evaluate bacterial recovery of *Pseudomonas aeruginosa* and *Staphylococcus aureus* from three different formulations of Cu surfaces. ATP bioluminescence (ATPB) and live-dead flow cytometry staining were performed in tandem to corroborate bacterial recovery and antimicrobial findings. While all three collection methods were able to recover bacteria, direct PF contact with Cu surface consistently exhibited 2–3 Log higher colony-forming units (CFU)/20-cm^2^ counts. No significant difference in bacterial counts was found when sponges or swabs were applied to Cu surfaces and inoculated onto 5% sheep blood agar plates (BAP). No difference was observed between PF and BAP counts when PF was used for recovery after indirect sample collection using Quick Swab. Comparison of microbial counts with live-dead staining and ATPB results suggests that the PF *direct* collection method revived stressed and dying bacteria. This phenomenon was not observed with the *indirect* PF method. Direct sampling of Cu surfaces using PF confers a survival advantage to bacteria stressed by Cu and may induce higher bacterial counts.

**IMPORTANCE:**

Self-sanitizing surfaces such as copper (Cu) are increasingly used on high-touch surfaces to prevent the spread of harmful viruses and bacteria. Being able to monitor the antimicrobial properties of Cu is fundamental in measuring its antimicrobial efficacy. Thorough investigations into reliable methods to enumerate bacteria from self-sanitizing surfaces are lacking in the literature. This study demonstrates that direct use of Petrifilm on Cu surfaces most likely revives stressed and dying bacteria, which induces increased bacterial counts. This phenomenon was not observed with indirect collection methods. Studies assessing time-kill kinetics or long-term efficacy of Cu should consider the impact of the collection method chosen.

## INTRODUCTION

Copper (Cu) is a well-established self-sanitizing metal capable of reducing microbial bioburden on its surfaces (1 – 3). Most recently, interest in its use has increased, in part, driven by the COVID-19 pandemic. The Environmental Protection Agency (EPA), ISO, and ASTM standards provide protocols to evaluate the bactericidal activity of Cu-containing products *in vitro* and recommend sonication or vortexing of the small coupon carriers (10–25 mm^2^) (4 – 6). However, the EPA and other regulatory bodies do not provide guidelines for testing of larger surfaces (6). Reviews of environmental sampling acknowledge the wide variation in sampling techniques *in vitro* and *in situ* but do not discuss the implications of this when evaluating self-sanitizing surfaces such as Cu (7 – 10).

Currently, environmental industrial microbiological testing (e.g., food services) employs direct and indirect contact sampling methods routinely and deems them to be equivalent (7, 9, 11). These sampling methods may not be applicable to self-sanitizing surfaces where the antimicrobial activity is continuous compared to that seen at a single point in time when disinfectants are used.

Measurement of the antimicrobial efficacy of self-sanitizing surfaces such as Cu could potentially be influenced by the collection method. This *in vitro* study assessed bacterial recovery of *Pseudomonas aeruginosa* and *Staphylococcus aureus* from three different types of Cu surfaces using Petrifilm (PF) aerobic plates applied *directly* to surfaces compared to two indirect collection devices: SurfACE Sponge-Stick premoistened cellulose sponges and 3M Quick Swab. In the event of a difference between direct and indirect methods, the secondary goal was to compare PF to 5% sheep’s blood agar plates (BAP) for bacterial enumeration when using an indirect collection method (3M Quick Swab).

## MATERIALS AND METHODS

### Bacterial strains and inoculum preparation

A modified version of the EPA protocol (4, 12) was used to prepare the inocula: *P. aeruginosa* (ATCC 15442) and *S. aureus* (ATCC 29213). Briefly, three to five isolated colonies were cultured in 20 mL of tryptic soy broth (TSB) (Oxoid, Canada) overnight to reach exponential growth phase. The following day, cultures were centrifuged and washed twice with phosphate-buffered saline (PBS), the supernatant was removed (for *P. aeruginosa*, the mucoid layer was removed from the cell pellet following the first centrifugation), and a 0.5 McFarland standard was prepared in 0.85% saline. The final preparation at a concentration of approximately 10^6^ colony-forming units (CFU)/mL included a soil load solution to mimic environmental bioburden. This consisted of 4-mg/mL mucin (SIGMA, USA), 5-mg/mL bovine serum albumin (Proliant Biologicals, USA), and 50-mg/mL yeast extract (SIGMA, USA) in sterile water and then filter sterilized. The final concentration of the inoculum was calculated by plating serial dilutions onto BAP and enumerating colonies after 48-hour incubation at 37°C. Additional inoculum dilutions (10^3^, 10^4^, and 10^5^ CFU/mL) were prepared for the direct collection method to ensure colonies were within countable ranges.

### Surfaces

Large sheets of surgical grade 316 stainless steel (SS) as the control and three commercially available Cu formulations were tested: (i) a solid-based formulation (80% Cu and 20% Ni), (ii) a thermal fabrication application on solid metal (80% Cu and 20% Ni), and (iii) a Cu-containing decal (91.3% Cu). All surfaces were examined to ensure they were intact prior to each experiment. Surfaces were permanently marked with standardized 20-cm^2^ circular areas for testing. Rectangular 20-cm^2^ areas were used for SurfACE Sponge-Stick premoistened cellulose sponges in neutralizing buffer (Romer Labs, Canada) to mimic the shape of the sponge for ease and efficiency of sampling. The surfaces were then soaked in Liquinox 1% (Alcanox Critical Cleaning Experts, USA) detergent for 2 hours to remove any grease/oil, subsequently rinsed thoroughly with sterile deionized water, and then dried with a lint-free wipe. Immediately prior to each experiment, the surfaces were sterilized by applying 95% ethanol for 10 min and allowed to air dry.

### Surface inoculation

A 100-µL aliquot of the bacterial inoculum preparation was evenly spread over each 20-cm^2^ rectangle or circle to within 1/8″ from the edge for the four surface types for each time point. Experiments with the sponges, Quick Swab (3M Canada, Canada), direct Aerobic Count PF (3M Canada, Canada), and ATP bioluminescence (ATPB) (Scigiene Corp., Canada) inoculation methods were performed in triplicate over three different days per organism (*n* = 9 replicates): For *P. aeruginosa*, the inoculum remained on surfaces for 0.5 and 1 hour, and for *S. aureus*, the inoculum remained on surface for 1 and 2 hours before sampling, as per the EPA recommendations and based on previous experience using a modified EPA protocol (4, 12). Time zero was defined as the moment the inoculum was plated onto each surface to account for drying inconsistencies between products. All surfaces remained uncovered during the experiment at 21°C at a relative humidity ranging from 50% to 54%.

### Sampling methods

**Fig 1:**
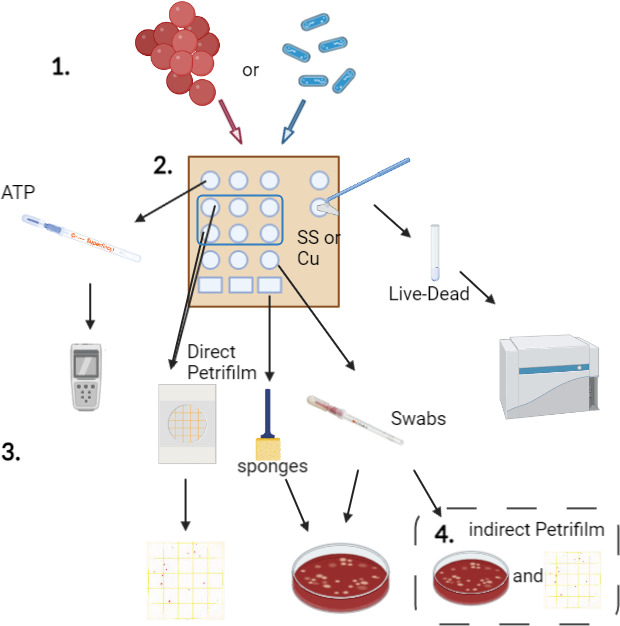
Graphical overview of enumeration methods. Either *S. aureus* or *P. aeruginosa* was inoculated onto four different surfaces. Bacteria were recovered using either Petrifilms, sponges, quickswabs, or cell scrapers for enumeration of viable bacteria. ATPB swabs were used to measure cleanliness and remaining active cellular material.

Following the appropriate time point, residual bacteria were collected as illustrated in Fig. 1.

#### Direct collection with PF aerobic count plate

As per the manufacturer’s instructions, the PF plates were re-hydrated with 1 mL of Letheen broth (Remel, USA) [10-g meat peptone, 5-g beef extract, 5-g sodium chloride, 0.7-g lecithin, and 5-g polysorbate 80 (TWEEN 80)/L of water] and allowed to set for 1 hour. At the times indicated in Section 2.3, the circular agar gel containing side of PF was pressed onto and just within the circular template followed by gentle rubbing of the entire circular area of the film with pad of finger for 10 seconds. Plates were incubated at 30°C for 48 hours. Enumeration was done using the 3M Petrifilm Plate Reader Advanced as well as being confirmed visually.

#### Indirect collection with 3M Quick Swab with subsequent inoculation onto BAP

Swabs were prewetted with 1 mL of Letheen-neutralizing broth before swabbing the circular template area vigorously for 20 seconds in three directions, holding the swab at a 30° angle and using even firm pressure while rotating the swabs as per manufacturer’s instructions. The swabs were then vortexed for 20 seconds to detach bacteria and the eluent expressed by squeezing the swab tip against the side of the tube. The 1 mL of bacteria-containing Letheen broth from the Quick Swab was used for multiple serial dilutions in Letheen broth and inoculated onto BAPs that were incubated at 37°C for 48 hours prior to enumeration. Enumeration was done manually using a colony counter.

#### Indirect collection with premoistened cellulose sponges with subsequent inoculation onto BAP

Similar to the method above, bacteria were retrieved from rectangular template areas at times previously described by rubbing the sponge vigorously on the rectangular template area using all parts of the sponge in the manufacturer’s specified sequence in both horizontal and vertical directions to maximize bacterial recovery. The sponge was then aseptically released from the handle into a sterile sample bag, 40 mL of sterile-deionized water was added, and the bag was then stomached with a Stomacher 400 Circulator (Seward, USA) for 1 min at 260 rpm. The liquid was aseptically transferred to a 50-mL tube while expressing as much liquid as possible from the sponge and then centrifuged for 15 min at 4,000 rpm. The supernatant was discarded, and the eluent of 2–3 mL (exact volume of eluent remaining measured) was used for the preparation of serial dilutions for inoculation onto BAP. The plates were incubated at 37°C, and colonies were counted after 48 hours. Enumeration was done manually using a colony counter.

### Evaluation of bacterial viability using live-dead stains

In tandem with methods in Section 2.4, 100-uL aliquots of *P. aeruginosa* and *S. aureus* inoculum were applied to the four different metal surfaces (SS, solid, spray-on, and decal Cu) onto 20-cm^2^ areas in duplicate per experiment and organism (*n* = 6). At predetermined times (0.5 and 1 hour for *P*. *aeruginos*, and 1 and 2 hours for *S. aureus*), bacteria were retrieved from the surfaces using PBS and a disposable cell scraper and transferred to tubes containing a mixture of SYTO 9 and propidium iodine (PI) (Invitrogen, CA, USA) fluorescent dyes to assess membrane damage and cell viability according to manufacturer’s instructions. Bacterial collection was timed for simultaneous collection of the two-time points to minimize the potential confounder of bacterial cell death that could occur between the two-time points. Bacteria were stained for 20 min at room temperature before analysis of the proportion of live (green staining), stressed (yellow stain), and dead (red staining) organisms using a CytoFlex flow cytometer with CytExpert Software version 2.4 (Beckman Coulter, USA). Fluorescein isothiocyanate (FITC) and PerCP-Cy5.5 filters were used for SYTO 9 and PI, respectively. Data were analyzed with Kaluza software version 2.1.3 (Beckman Coulter, USA).

### ATPB assay

As an additional measure of bioburden, ATPB Hygiena SuperSnaps (Scigiene, USA) were used in parallel in a predefined inoculated area separate from the PF, swabs, and sponges according to manufacturer’s instruction. A calibrated EnSUREV2 ATPB Luminometer (Hygiena, USA) was used to read bioluminescence in relative light units (RLU). SuperSnap swabs were used to collect inoculum from areas at the time periods as previously described. A negative control for each surface was included. Swabs were held at a 30° angle to the circular template surface and swabbed vigorously for 20 seconds in two directions while rotating as per manufacturer’s instructions.

### Comparison of counts for 3M Quick Swab collection and plating on PF and BAP

To confirm if the differences noted between the direct and indirect collection methods were due to the composition of the PF, a second experiment was performed (Fig. 1, Part 4). *P. aeruginosa* and *S. aureus* (at 0.5 and 1 hour, respectively) were collected in 1 mL of Letheen broth using a 3M Quick Swab that was subsequently divided and plated onto both Petrifilm and BAP plates. The same bacterial strains, inoculum preparation, surfaces, and surface preparation were used as in Sections 2.1 and 2.3. PF plates were incubated at 30°C, and colony counts were recorded at 24 and 48 hours. Inoculated BAPs were incubated at 37°C, and colony counts were performed at 48 hours. Enumeration was done manually using a colony counter for BAPs while PF plates were read with the 3M Petrifilm Plate Reader Advanced as well as confirmed visually. For each organism, results were done in triplicate in one separate experiment (*n* = 3).

### Statistical analysis

Results and error bars are presented as ± standard deviation (SD). To calculate Log_10_ reduction, zeros were substituted for results less than 0.5 for geometric mean values. The recovery percentage was calculated as the ratio of the Log mean CFU recovered from inoculated surfaces to the Log mean CFU from the inoculum used. Two-way analysis of variance (ANOVA) with Tukey’s multiple comparisons test or Kruskal-Wallis test with Dunn’s multiple comparisons test was applied to determine statistical differences between collection methods. Significance was set as *P < 0.05, **P < 0.01, ***P < 0.001, and ****P < 0.0001. Data were analyzed using GraphPad Prism version 9.1.0 (GraphPad Software, USA).

## RESULTS

### Comparison of collection methods

Direct PF results for *P. aeruginosa* on Cu surfaces exhibited a range of percent recovery between 48.1% and 107.6% at both 0.5 and 1 hour (Table 1). SS controls showed no antimicrobial efficacy with a percent recovery of 118.8%–121.8% at 0.5–1 h. Direct PF collection for *S. aureus* ranged from a recovery of 57.2%–90.3% for Cu products and 100.7–102.6% for SS controls at 1 and 2 hours.

**TABLE 1:** Comparison of number of viable *P. aeruginosa* (ATCC 15442) and *S. aureus* (ATCC 29213) bacteria collected using 3M Petrifilm plates, 3M Quick Swab, and cellulose SurfACE sponges from stainless steel and Cu products for *n* = 9 per organism^*a*^

Sampling methods/products	Petrifilm aerobic plates				3M swabs				Cellulose sponge			
	Log_10_ CFU^*c*^	SD^*e*^	Log_10_ difference	% Recovery^*b*^	Log_10_ CFU	SD	Log_10_ difference	% Recovery^*b*^	Log_10_ CFU	SD	Log_10_ difference	% Recovery^*b*^
*P. aeruginosa*, 0.5 hour												
SS	5.49	4.73	−	121.8	5.36	4.75	–	104.0	6.09	5.39	−	117.3
Decal	4.62	4.10	0.87	106.9	4.00	3.57	1.36	32.7	2.56	2.20	3.54	53.8
Thermal fabrication	2.02	1.96	3.47	48.1	0	0	5.36	0.0	0	0	6.09	0.0
Integral	3.13	3.15	2.36	52.9	0.28	0.44	5.09	0.0	1.64	1.19	4.45	18.3
*P. aeruginosa,* 1 hour												
SS	5.27	4.58	−	118.8	5.16	4.84	−	107.4	5.59	4.83	−	98.0
Decal	4.54	3.75	0.73	107.6	0.05	0.28	5.12	0.0	0	0	5.59	0.0
Thermal fabrication	1.93	1.74	3.34	51.5	0	0	5.16	0.0	0	0	5.59	0.0
Integral	1.93	1.73	3.34	48.7	0	0	5.16	0.0	0	0	5.59	0.0
*S. aureus,* 1 hour												
SS	5.67	5.39	−	102.6	5.55	5.34	−	89.8	5.74	5.38	−	94.5
Decal	5.31	4.26	0.36	82.6	4.31	4.32	1.24	34.0	2.34	2.37	3.40	45.6
Thermal fabrication	3.61	2.83	2.06	66.3	0.00	0.00	5.55	0.0	1.79	1.67	3.95	34.8
Integral	3.45	3.05	2.23	57.2	0.28	0.44	5.28	3.1	2.64	2.73	3.10	42.6
*S. aureus,* 2 hours												
SS	5.51	4.82	−	100.7	5.40	5.05	−	87.7	5.63	5.12	−	91.8
Decal	5.30	5.03	0.21	90.3	1.34	1.36	4.07	18.0	1.68	1.51	3.95	38.2
Thermal fabrication	3.30	2.96	2.21	59.9	0.28	0.48	5.13	1.5	1.97	1.89	3.66	37.6
Integral	3.52	3.16	2.00	67.0	0.99	0.93	4.41	12.1	2.40	2.34	3.23	42.4

^*a*^ Inoculum CFU/mL mean ± SD: 1.1 × 10^6^ ± 7.1 × 10^5^ for *P. aeruginosa* and 4.1 × 10^7^ ± 6.1 × 10^7^ for *S. aureus*.

^*b*^ % Recovery was calculated as the ratio Log_10_ CFU surface to Log_10_ inoculum/100µL.

^*c*^ CFU/20-cm^2^ carrier area.

^*d*^ SS, stainless steel.

^*e*^ SD, standard deviation.

In contrast, indirect collection using both Quick Swab and sponges for *P. aeruginosa* exhibited between 0% and 53.8% recovery for Cu products and between 98% and 117.3% for SS controls. Ranges for indirect collection of *S. aureus* yielded 0%–45.6% recovery for Cu products and 87.7%–94.5% for SS. Overall, the average percent recovery of *P. aeruginosa* and *S. aureus* compared to the inoculum was higher for samples collected using the direct PF method compared to indirect sponges or swabs, regardless of the Cu surface composition.

**Fig 2:**
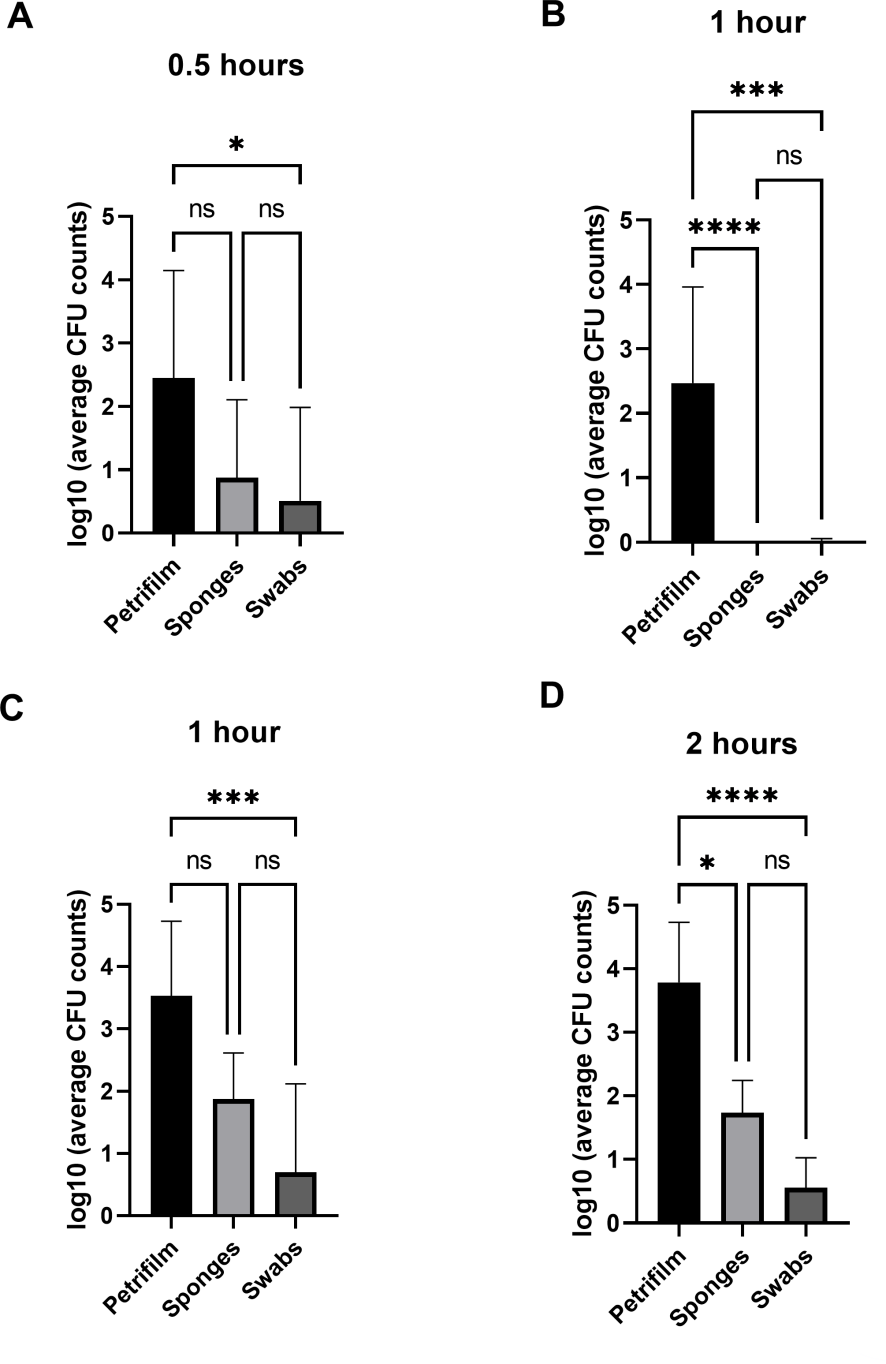
Comparison of collection methods from all copper products between (A and B) *P. aeruginosa* and (C and D) *S. aureus* (±SD, *n* = 9). *P < 0.05, **P < 0.01, ***P < 0.001, and ****P < 0.0001.

To determine whether the collection methods were statistically different on self-sanitizing Cu surfaces, we pooled the Log_10_ CFU counts for all three Cu formulations. At 0.5 hour, Log_10_ CFU counts for *P. aeruginosa* from PF used directly on surfaces were significantly higher than the indirect swab method (*P* = 0.002) and trended to be higher than the indirect sponge method (*P* = 0.12) (Fig. 2A). At 1 hour, Log_10_ CFU *P. aeruginosa* counts on PF were significantly higher than both sponges and swabs (*P* ≤ 0.0001 and *P* = 0.0002), respectively (Fig. 2B). Similarly, the direct PF method consistently exhibited Log_10_ CFU values for *S. aureus* that were significantly higher compared to sponges at 2 hours (*P* = 0.031) and swabs (*P* = 0.003 and *P* < 0.0001) at 1 and 2 hours, respectively (Fig. 2C and D). No statistical difference was observed between sponges and Quick Swab for both *S. aureus* and *P. aeruginosa*.

### ATPB

**Fig 3:**
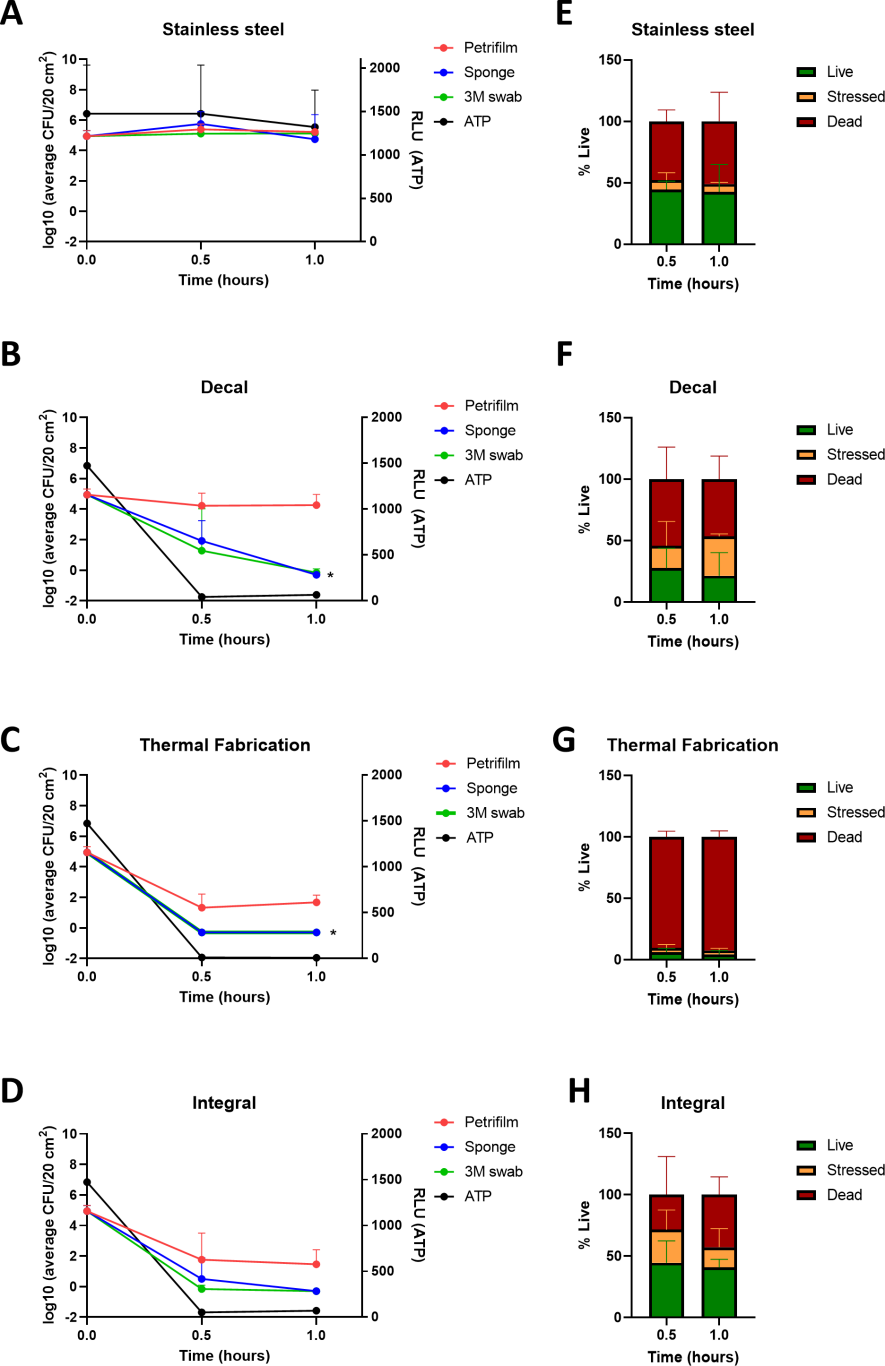
Time-kill analysis comparing three surface collection methods on different copper formulations for *P. aeruginosa.* (**A–D**) Log_10_ of average colony-forming units (CFU) (left *y*-axis) and ATPB relative light units (RLU) (right *y*-axis) of different bacterial collection methods on stainless steel, copper decal, thermal fabrication, and integral copper, at 0.5- and 1-hour post-inoculation, respectively (±SD, *n =* 9). (**E–H**) Live-dead percentages taken from (E) SS, (**F**) decal, (**G**) thermal fabrication, and (H) integral copper surfaces (*n* = 6). * indicates statistical significance compared to Petrifilm at matched time point. *P < 0.05.

**Fig 4:**
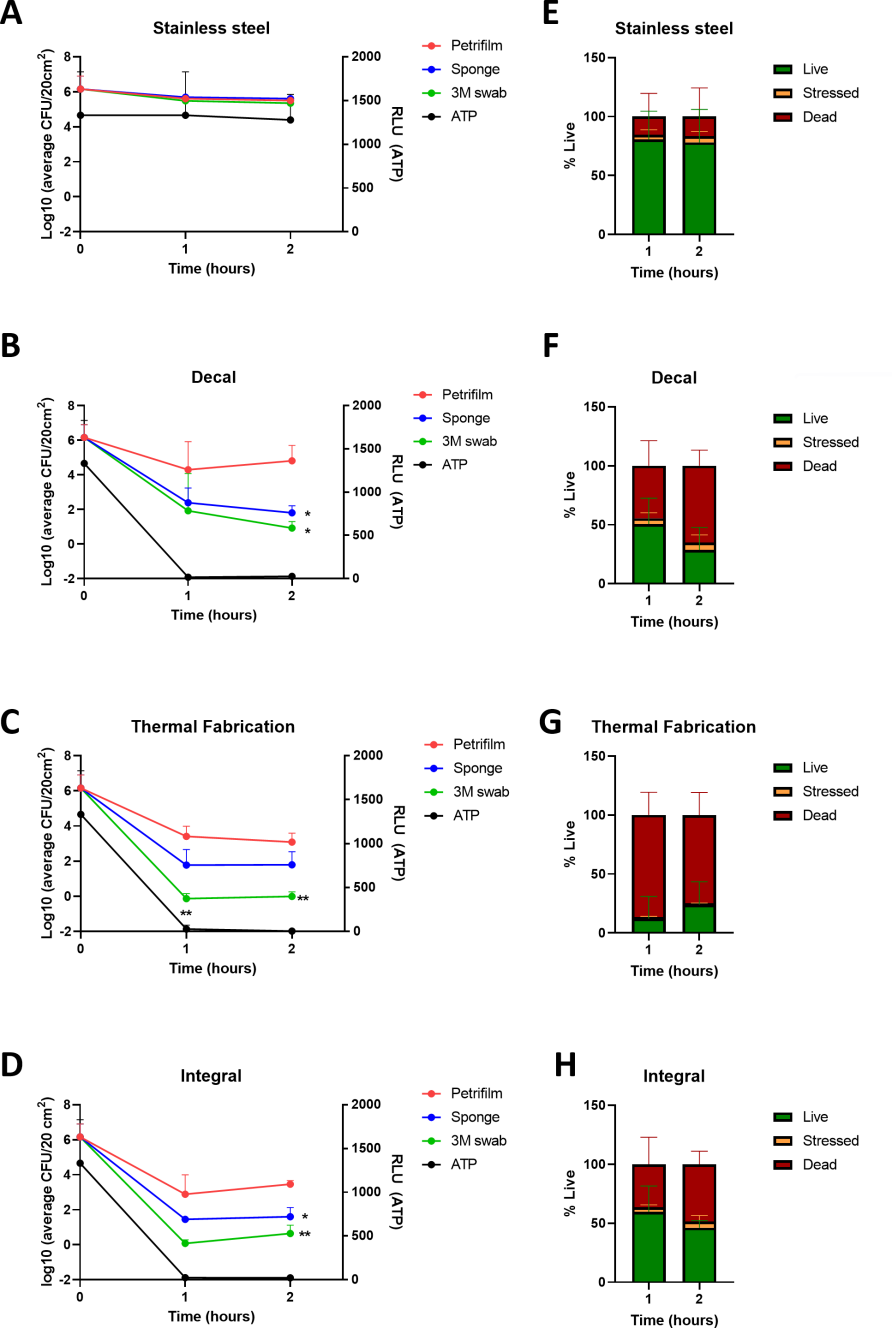
Time-kill analysis comparing three surface collection methods on different copper formulations for *S. aureus.* (**A–D**) Log_10_ of average colony-forming units (CFU) (left *y*-axis) and ATPB relative light units (RLU) (right *y*-axis) of different bacterial collection methods on stainless steel, copper decal, thermal fabrication, and integral copper, at 1- and 2-hour post-inoculation, respectively (±SD, *n =* 9). (**E–H**) Live-dead percentages taken from (E) SS, (**F**) decal, (**G**) thermal fabrication, and (H) integral copper surfaces (*n* = 6). * indicates statistical significance compared to Petrifilm at matched time point. *P < 0.05 and **P < 0.01.

Following the important observation that different collection methods impacted bacterial recovery over time, the ATPB readings were compared to the collection methods using time-kill analysis. For both *P. aeruginosa* (Fig. 3A through D; Table S1) and *S. aureus* (Fig. 4A through D; Table S1), ATPB readings followed similar kinetics to indirect collection methods. Readings ranged between 7 and 76 RLU/20 cm^2^ for Cu formulations for *P. aeruginosa* compared to 1,300–1,500 RLU/20 cm^2^ for SS for all Cu formulations at 0.5 and 1 hour. Cu RLU readings trended to be lower for *S. aureus* with a range of 1–31 RLU/20 cm^2^ and SS 1,300 RLU/20 cm^2^ at 1 and 2 hours.

### Live-dead staining

The live-dead staining that occurred concurrently with the previous collection methods was analyzed to determine whether the discrepancies between direct and indirect collection methods were inferring an advantage to stressed and dying bacteria. In tandem with the collection methods, live-dead staining on collected bacteria was performed to determine the percentage of live (green), stressed (yellow), and dead (red) bacteria (Fig. S1). Stressed bacteria were defined as organisms that co-stained for the two dyes, an indication of compromised membrane integrity. Approximately 45% of the *P. aeruginosa* inoculum was viable on SS after 30 min and 1 hour (Fig. 3E) compared to Cu formulations that demonstrated a reduced viability of 26% at 0.5 hour (range 6.3–44.5%) and 22% at 1 hour (range 4.4–40.8%) (Fig. 3F through H). A significant amount (79%) of the *S. aureus* inoculum was still viable on SS after 1 and 2 hours (Fig. 4E) compared to the Cu formulations that demonstrated a reduced viability of an average of 41% (range 12.6–59.6%) at 1 hour and 32.9% (range 24.1–46.2%) at 2 hours for *S. aureus* (Fig. 4E through H). The live-dead percent viability for *P. aeruginosa* (Fig. 3E through H) and *S. aureus* (Fig. 4E through H) was compared to the Log_10_ CFU/20-cm^2^ counts for each Cu substrate (Fig. 3A through D and Fig. 4A through D). Direct PF colony counts were observed to be higher than the percentviability observed by flow cytometry, whereas indirect methods were comparable.

### Direct vs indirect use of PF

Based on the discrepancies in percent recovery (Table 1) and live-dead staining observed, it was hypothesized that direct collection by PF could infer an advantage to stressed and dying bacteria. To determine if this effect was due to the collection method versus the composition of the media, PF was compared to BAP post-indirect collection. When PF was inoculated after indirect collection using a swab, the results were similar to those using BAP (Fig. 1, Section 4). There was no significant difference between mean CFU and percent recovery for BAP compared to PF counts for all surfaces when the collection was made by Quick Swab (Table 2).

**TABLE 2:** Comparison of viable *P. aeruginosa* (ATCC 15442) and *S. aureus* (ATCC 29213) colonies from 5% sheep BAP and Petrifilm aerobic count plates collected from the surfaces with 3M Quick Swab^*a*^

Surface products	Petrifilm aerobic plates				BAP			
	Mean CFU^*b*^	SD^*c*^	Log_10_ difference	% Recovery	Mean CFU^*b*^	SD	Log_10_ difference	% Recovery
*P. aeruginosa*, 0.5 hour								
SS	5.18	5.00	−	81.9	5.08	4.43	−	80.1
Decal	1.45	1.41	3.73	22.9	1.48	1.68	3.60	23.3
Thermal fabrication	0.00	0.00	5.18	0	0.11	0.36	4.97	2
Integral	0.72	0.79	4.45	0	0.00	0.00	5.08	0
*S. aureus*, 1 hour								
SS^*d*^	5.32	4.88	−	89.7	5.32	4.97	−	84.1
Decal	2.71	2.85	2.61	45.3	2.18	1.65	3.15	34.2
Thermal fabrication	2.04	1.00	3.28	34.8	1.96	1.36	3.36	30.3
Integral	1.85	1.68	3.48	35	2.00	1.87	3.32	38

^*a*^ % Recovery was calculated as the ratio Log_10_ CFU surface to Log_10_ inoculum. Inoculum CFU/100 µL: 2.1 × 10^6^ for *P. aeruginosa* and 9.6 × 10^5^ for *S. aureus*.

^*b*^ Mean CFU = CFU/20 cm^2^ surface area.

^*c*^ SD, standard deviation.

^*d*^ SS, stainless steel.

## DISCUSSION

The ISO and EPA protocols or their modified versions (4, 6, 12) have been developed for controlled environments using small coupon carriers and are impractical for use in real-life settings. Sampling in the field is important in determining antimicrobial activity under real-life use, but the method of bacterial collection is not standardized. In particular, the differences in bacterial retrieval using either direct media (contact) plating and indirect (swab or sponge) methods have not been well characterized. In this experiment, different bacterial collection methods that could be used *in situ* to assess self-sanitizing Cu surfaces were evaluated. PF is a well-established standard for microbial enumeration in the food and environmental industry and has also been used to evaluate the antimicrobial efficacy of self-disinfecting surfaces (7, 13, 14). However, preliminary results from an ongoing in-use experiment being conducted by the authors found discrepancies in the expected reductions in CFUs when direct contact of PF to surfaces was used (unpublished data). This led to the formal study evaluating the different collection methods described in this article.

This simple yet telling study clearly shows that the sampling methodology has a direct impact on the number of bacteria recovered on self-sanitizing surfaces. The direct PF collection demonstrated a significantly higher percent recovery compared to indirect methods for both *P. aeruginosa* and *S. aureus* from all Cu formulations that were not observed on SS.

These findings were consistent with the secondary use of ATPB that was used to support the results of the bacterial collection studies. The use of ATPB as a measure of bactericidal activity on surfaces is controversial (15 – 21) with some articles finding good correlation between RLU and other bacterial outputs such as aerobic colony counts and pyogenic or endotoxin activity (18, 19) in laboratory and hospital settings. Others have reported good reproducibility of CFU and ATPB readings but poor correlative readings (16, 17, 20) in healthcare settings. This experiment demonstrated that the ATPB readings for *S. aureus* and *P. aeruginosa* consistently reflected the kinetics of the colony counts on Cu surfaces for indirect collection methods.

The live-dead staining analysis further corroborated the discrepancy observed with the expected percent recovery and Log_10_ CFU counts for direct and indirect collection methods. Live-dead data found on average 22%–26% surviving *P. aeruginosa* and 32.9%–41% surviving *S. aureus*, both of which were considerably lower compared to the 48.1%–107.6% recovery for both *P. aeruginosa* and *S. aureus* using direct PF collection on Cu surfaces. It was further noted that stressed bacteria were more apparent on Cu surfaces inoculated with *P. aeruginosa* compared to *S. aureus*. This may be due to the high bactericidal efficacy of Cu against Gram-negative bacteria corresponding to more rapid cell envelope damage due to their thinner peptidoglycan cell wall (22, 23). Recent articles have reported conflicting evidence on the threshold for live-dead staining of bacteria (24, 25). In one paper, the authors report that yellow fluorescing bacteria should be classified as dead (24), while anothers suggested that anoxic bacteria or ones with high membrane potential can uptake the dead cell dye propidium iodide, while still being viable (25). However, both findings were based on microscopic observations, which are unable to distinguish between the intermediate levels of co-staining or yellow fluorescence. Therefore, bacteria co-expressing red and green viability dyes were considered as intermediate states of membrane damage or “stressed” as per flow cytometry live-dead literature (26). However, it is important to note that live-dead staining should be used as a complementary assessment, as the permeability of bacterial cells could be transient to fluorescent dye uptake (2).

It has been reported that one method of action for Cu’s bactericidal properties is through the release of Cu ions, which upon uptake by bacteria produce reactive oxygen species that then induce cell membrane damage (27, 28). Therefore, collection methods with chelating and quenching agents could inhibit the release of Cu ions and neutralize Cu’s bactericidal activity. Correspondence with 3M has confirmed the absence of chelating agents in PF aerobic count proprietary media confirming that the aberrant counts observed in this study are not due to media composition effects. Consequently, these findings suggest that direct collection by contact plating onto PF may revive stressed bacteria. This theory was supported by the use of the indirect 3M Quick Swab to collect bacteria followed by plating onto PF and BAPs.

For this study, all surfaces were subjected to the same degreasing and rinsing steps with 1% Liquinox. As Liquinox is removed with rinsing for a residue-free surface, all detergents and disinfectants would be nominal on the surfaces and would not require neutralization with buffers. Therefore, although different collection media were used (Letheen broth, neutralizing buffer, or PBS), this would not affect the collection of bacteria from surfaces. Indeed, for live-dead staining, which did not require additional culturing steps, PBS was utilized for enhanced resolution through the flow cytometer. Other procedural factors should also be considered when comparing the collection methods and how this might affect the recovery of bacteria from SS and Cu surfaces. Indirect collection methods involve more mechanical manipulation such as stomaching and centrifugation for sponges, scraping for live-dead staining, and shaking and squeezing of swabs. As indirect methods involve some manipulation of the collection device to disassociate the bacteria, this could contribute to a decreased survivability compared to the direct plating method for already stressed and compromised bacteria collected from Cu surfaces. However, no recovery discrepancy was observed on SS using indirect methods (Fig. 3A and 4A), which suggests that there are nominal differences between the collection procedures on non-self-sanitizing surfaces such as Cu. For more common surfaces such as plastic or SS, the use of the direct plating method may or may not better reflect the effect of hands touching a surface and additional studies are required to address this question.

Previous studies have used PF for Cu time-kill studies (29 – 32); however, they have used the indirect spread plate technique as opposed to direct collection (13, 14). Reed et al. used a direct sampling method with *Escherichia coli* on Cu discs and found Cu to be bactericidal. However, the Cu discs were kept in contact with PF for the length of the experiment as opposed to the collection at set time points, and a bacterium more susceptible to Cu than *S. aureus* was used. Prolonged contact could prevent stressed bacterial populations from reviving, however, this method is impractical in real-life settings. Therefore, direct plating using PF on Cu surfaces may result in an underestimation of the efficacy of Cu as an antimicrobial.

There are several limitations involved in this study. Firstly, the mucoid layer on *P. aeruginosa* prior to inoculation was removed in order to determine the bacterial concentration using McFarland standards as per EPA protocol. In effect, while comparable CFU counts from SS to the inoculum were observed, viability from live-dead staining of *P. aeruginosa* was reduced. This could potentially be caused by increased desiccation on surfaces (33). Variations in drying time of the inoculum and the ability to spread the inoculum on the different surfaces were observed. The experiment was performed in a controlled laboratory environment with laboratory strains. While this enabled a thorough and controlled investigation of the various collection methods, it did not account for variance in strains and resistance genes that could occur in real-life scenarios. Further studies are required to examine the effect of these collection methods *in situ* on Cu surfaces.

### Conclusion

This study revealed that sampling methodology is crucial when evaluating the antimicrobial efficacy of self-sanitizing surfaces such as Cu. The use of PF allows for user-friendly, automatable, efficient, standardized, and reliable environmental sampling. However, direct plating with PF may cause a decrease in the measured antibacterial efficacy of Cu surfaces by potentially reviving stressed populations of bacteria. Finally, ATPB using SuperSnaps is a quick complimentary test for measuring antimicrobial efficacy on self-sanitizing surfaces.

## Data Availability

The raw data used to prepare the figures are available upon request. The copper surfaces are commercially available in Canada and PMRA registered by the following manufacturers: Coptek, Aereus, and Trimco.

## SUPPLEMENTAL MATERIAL

Fig. S1 spectrum.02441-23-s0001.tif Flow cytometry live-dead results as a measure of antibacterial effect of copper.

Table S1 spectrum.02441-23-s0002.docx Comparison of ATPB readings versus collection methods for both *P. aeruginosa* and *S. aureus*.

ASM does not own the copyrights to Supplemental Material that may be linked to, or accessed through, an article. The authors have granted ASM a non-exclusive, world-wide license to publish the Supplemental Material files. Please contact the corresponding author directly for reuse.

## References

[1] EPA . 2008. Antimicrobial copper alloys—group V. Programs OoP, U.S. Environmental Protection Agency

[2] Salah I , Parkin IP , Allan E . 2021. Copper as an antimicrobial agent: recent advances. RSC Adv 11:18179–18186. doi:10.1039/d1ra02149d 35480904 PMC9033467

[3] Colin M , Klingelschmitt F , Charpentier E , Josse J , Kanagaratnam L , De Champs C , Gangloff SC . 2018. Copper alloy touch surfaces in healthcare facilities: an effective solution to prevent bacterial spreading. Materials (Basel) 11:2479. doi:10.3390/ma11122479 30563265 PMC6317222

[4] EPA . 2016. Protocol for the evaluation of bactericidal activity of hard, non-porous copper containing surface products, on Environmental Protection Agency. Available from: https://www.epa.gov/pesticide-registration/updated-draft-protocol-evaluation-bactericidal-activity-hard-non-porous. Retrieved 27 Sep 2016.

[5] ASTM-E2197 . 2018. Standard quantitative disk carrier test method for determining bactericidal, virucidal, fungicidal, mycobactericidal, and sporicidal activities of chemical. ASTM Int.

[6] ISO . 2022. Method for the evaluation of basic bactericidal activity of a non-porous surface.

[7] Chai J , Donnelly T , Wong T , Bryce EA . 2013. Environmental sampling of hospital surfaces: assessing methodological quality. Can J Infect Control 33:138–145.

[8] Chyderiotis S , Legeay C , Verjat-Trannoy D , Le Gallou F , Astagneau P , Lepelletier D . 2018. New insights on antimicrobial efficacy of copper surfaces in the healthcare environment: a systematic review. Clin Microbiol Infect 24:1130–1138. doi:10.1016/j.cmi.2018.03.034 29605564

[9] Ismaïl R , Aviat F , Michel V , Le Bayon I , Gay-Perret P , Kutnik M , Fédérighi M . 2013. Methods for recovering microorganisms from solid surfaces used in the food industry: a review of the literature. Int J Environ Res Public Health 10:6169–6183. doi:10.3390/ijerph10116169 24240728 PMC3863893

[10] Ríos-Castillo AG , Ripolles-Avila C , Rodríguez-Jerez JJ . 2021. Evaluation of bacterial population using multiple sampling methods and the identification of bacteria detected on supermarket food contact surfaces. Food Control 119:107471. doi:10.1016/j.foodcont.2020.107471

[11] van de Lagemaat M , Grotenhuis A , van de Belt-Gritter B , Roest S , Loontjens TJA , Busscher HJ , van der Mei HC , Ren Y . 2017. Comparison of methods to evaluate bacterial contact-killing materials. Acta Biomater 59:139–147. doi:10.1016/j.actbio.2017.06.042 28666886

[12] Bryce EA , Velapatino B , Akbari Khorami H , Donnelly-Pierce T , Wong T , Dixon R , Asselin E . 2020. In vitro evaluation of antimicrobial efficacy and durability of three copper surfaces used in healthcare. Biointerphases 15:011005. doi:10.1116/1.5134676 32041413

[13] Reed JH , Gonsalves AE , Román JK , Oh J , Cha H , Dana CE , Toc M , Hong S , Hoffman JB , Andrade JE , Jo KD , Alleyne M , Miljkovic N , Cropek DM . 2019. Ultrascalable multifunctional nanoengineered copper and aluminum for antiadhesion and bactericidal applications. ACS Appl Bio Mater 2:2726–2737. doi:10.1021/acsabm.8b00765 35030808

[14] Mikolay A , Huggett S , Tikana L , Grass G , Braun J , Nies DH . 2010. Survival of bacteria on metallic copper surfaces in a hospital trial. Appl Microbiol Biotechnol 87:1875–1879. doi:10.1007/s00253-010-2640-1 20449737

[15] Moore G , Griffith C . 2002. A comparison of traditional and recently developed methods for monitoring surface hygiene within the food industry: an industry trial. Int J Environ Health Res 12:317–329. doi:10.1080/0960312021000056429 12590780

[16] Olafsdottir LB , Wright SB , Smithey A , Heroux R , Hirsch EB , Chen A , Lane B , Sawhney MS , Snyder GM . 2017. Adenosine triphosphate quantification correlates poorly with microbial contamination of duodenoscopes. Infect Control Hosp Epidemiol 38:678–684. doi:10.1017/ice.2017.58 28414009

[17] Snyder GM , Holyoak AD , Leary KE , Sullivan BF , Davis RB , Wright SB . 2013. Effectiveness of visual inspection compared with non-microbiologic methods to determine the thoroughness of post-discharge cleaning. Antimicrob Resist Infect Control 2:26. doi:10.1186/2047-2994-2-26 24088298 PMC3852477

[18] Deshpande A , Dunn AN , Fox J , Cadnum JL , Mana TSC , Jencson A , Fraser TG , Donskey CJ , Gordon SM . 2020. Monitoring the effectiveness of daily cleaning practices in an intensive care unit (ICU) setting using an adenosine triphosphate (ATP) bioluminescence assay. Am J Infect Control 48:757–760. doi:10.1016/j.ajic.2019.11.031 31883729

[19] Liebers V , Bachmann D , Franke G , Freundt S , Stubel H , Düser M , Kendzia B , Böckler M , Brüning T , Raulf M . 2015. Determination of ATP-activity as a useful tool for monitoring microbial load in aqueous humidifier samples. Int J Hyg Environ Health 218:246–253. doi:10.1016/j.ijheh.2014.11.004 25535006

[20] van Arkel A , Willemsen I , Kluytmans J . 2021. The correlation between ATP measurement and microbial contamination of inanimate surfaces. Antimicrob Resist Infect Control 10:116. doi:10.1186/s13756-021-00981-0 34362450 PMC8349058

[21] Finger S , Wiegand C , Buschmann H-J , Hipler U-C . 2012. Antimicrobial properties of cyclodextrin–antiseptics-complexes determined by microplate laser nephelometry and ATP bioluminescence assay. Int J Pharm 436:851–856. doi:10.1016/j.ijpharm.2012.07.009 22877865

[22] Espírito Santo C , Lam EW , Elowsky CG , Quaranta D , Domaille DW , Chang CJ , Grass G . 2011. Bacterial killing by dry metallic copper surfaces. Appl Environ Microbiol 77:794–802. doi:10.1128/AEM.01599-10 21148701 PMC3028699

[23] Silhavy TJ , Kahne D , Walker S . 2010. The bacterial cell envelope. Cold Spring Harb Perspect Biol 2:a000414. doi:10.1101/cshperspect.a000414 20452953 PMC2857177

[24] Stiefel P , Schmidt-Emrich S , Maniura-Weber K , Ren Q . 2015. Critical aspects of using bacterial cell viability assays with the fluorophores SYTO 9 and propidium iodide. BMC Microbiol 15:36. doi:10.1186/s12866-015-0376-x 25881030 PMC4337318

[25] Kirchhoff C , Cypionka H . 2017. Propidium ion enters viable cells with high membrane potential during live-dead staining. J Microbiol Methods 142:79–82. doi:10.1016/j.mimet.2017.09.011 28927972

[26] Berney M , Hammes F , Bosshard F , Weilenmann H-U , Egli T . 2007. Assessment and interpretation of bacterial viability by using the LIVE/DEAD BacLight kit in combination with flow cytometry. Appl Environ Microbiol 73:3283–3290. doi:10.1128/AEM.02750-06 17384309 PMC1907116

[27] Grass G , Rensing C , Solioz M . 2011. Metallic copper as an antimicrobial surface. Appl Environ Microbiol 77:1541–1547. doi:10.1128/AEM.02766-10 21193661 PMC3067274

[28] Lee I-C , Ko J-W , Park S-H , Lim J-O , Shin I-S , Moon C , Kim S-H , Heo J-D , Kim J-C . 2016. Comparative toxicity and biodistribution of copper nanoparticles and cupric ions in rats. Int J Nanomedicine 11:2883–2900. doi:10.2147/IJN.S106346 27366066 PMC4913985

[29] Lee J-S , Lee Y-S , Kim M-S , Hyun S-K , Kang C-H , So J-S , Yoon E-H . 2013. Antibacterial characteristics of lotus-type porous copper. Adv Mater Sci Eng 2013:1–4. doi:10.1155/2013/608350

[30] Lee I , Cheon HJ , Adhikari MD , Tran TD , Yeon K-M , Kim MI , Kim J . 2020. Glucose oxidase-copper hybrid nanoflowers embedded with magnetic nanoparticles as an effective antibacterial agent. Int J Biol Macromol 155:1520–1531. doi:10.1016/j.ijbiomac.2019.11.129 31751699

[31] Klein TY , Wehling J , Treccani L , Rezwan K . 2013. Effective bacterial inactivation and removal of copper by porous ceramics with high surface area. Environ Sci Technol 47:1065–1072. doi:10.1021/es3045828 23273049

[32] Nakhaie D , Williams TC , Velapatino B , Bryce EA , Charles MK , Asselin E , Clifford AM . 2022. An engineered nanocomposite copper coating with enhanced antibacterial efficacy. Adv Materials Inter 9:2201009. doi:10.1002/admi.202201009

[33] Davies D . 2003. Understanding biofilm resistance to antibacterial agents. Nat Rev Drug Discov 2:114–122. doi:10.1038/nrd1008 12563302

