# Correction for Williams et al., “*In vitro* comparison of methods for sampling copper-based antimicrobial surfaces”

**DOI:** 10.1128/spectrum.00315-24

**Published:** 2024-03-19

**Authors:** T. C. Williams, T. Woznow, B. Velapatino, E. Asselin, D. Nakhaie, E. A. Bryce, M. Charles

## AUTHOR CORRECTION

Volume 11, no. 6, e02441-23, 2023, https://doi.org/10.1128/spectrum.02441-23. In the first sentence of the “Surfaces” paragraph of the Materials and Methods section, “(ii) a thermal fabrication application on solid metal (80% Cu and 20% Ni)” should read “(ii) a thermal fabrication application on solid metal (approximately 67% Cu and remaining Ni).”

