# Supplementary figures and images for: In vitro comparison of methods for sampling copper-based antimicrobial surfaces
Source: Microbiol Spectr. 2023 Oct 17;11(6):e02441-23. doi: 10.1128/spectrum.02441-23 (PMC10714924; doi:10.1128/spectrum.02441-23)

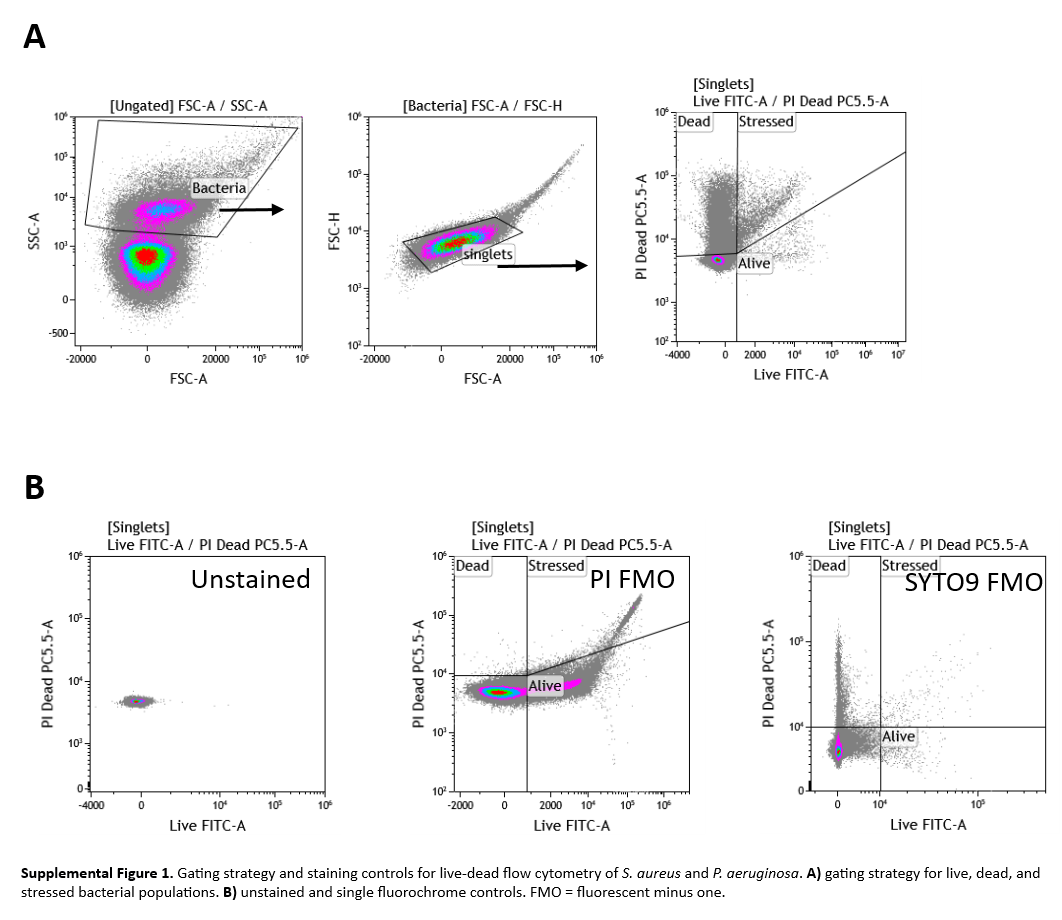

Supplement: Fig. S1 — Flow cytometry live-dead results as a measure of antibacterial effect of copper. [file spectrum.02441-23-s0001.tif]
