# Supplementary material for: In vitro comparison of methods for sampling copper-based antimicrobial surfaces
Source: Microbiol Spectr. 2023 Oct 17;11(6):e02441-23. doi: 10.1128/spectrum.02441-23 (PMC10714924; doi:10.1128/spectrum.02441-23)
Supplement: Table S1 — Comparison of ATPB readings versus collection methods for both P. aeruginosa and S. aureus. [file spectrum.02441-23-s0002.docx]

**Supplemental Tables**

**Supplemental Table 1:** *Pseudomonas aeruginosa* (ATCC 15442) and *Staphylococcus aureus* (ATCC 29213) organic material on SS and Cu products collected using ATP Bioluminescence swabs.

| **Surface Products** | **ATP Bioluminescence SuperSnap swabs** | | | | |
| --- | --- | --- | --- | --- | --- |
|  | **Mean (RLU)/20cm^2^** | | **SD** | **% Reduction** | **% Recovery** |
|  | ***Pseudomonas aeruginosa*, 0.5 h** | | | | |
| SS | 1500 | 280 | |  |  |
| Decal | 47 | 22 | | 96.7 | 3.1 |
| Thermal Fabrication | 14 | 8 | | 99.1 | 0.9 |
| Integral | 54 | 10 | | 95.9 | 3.6 |
|  | ***Pseudomonas aeruginosa***, 1 h | | | | |
| SS | 1300 | 190 | |  |  |
| Decal | 66 | 10 | | 94.6 | 5.0 |
| Thermal Fabrication | 7 | 1 | | 99.4 | 0.6 |
| Integral | 76 | 30 | | 94.2 | 5.7 |
|  | ***Staphylococcus aureus*, 1 h** | | | | |
| SS | 1300 | 180 | |  |  |
| Decal | 21 | 15 | | 98.8 | 1.5 |
| Thermal Fabrication | 1 | 1 | | 99.9 | 0.1 |
| Integral | 27 | 15 | | 98.0 | 2.0 |
|  | ***Staphylococcus aureus*, 2 h** | | | | |
| SS | 1300 | 150 | |  |  |
| Decal | 31 | 19 | | 97.7 | 2.4 |
| Thermal Fabrication | 6 | 6 | | 99.7 | 0.4 |
| Integral | 28 | 18 | | 98.2 | 2.2 |

Inoculum CFU mean ± SD: 1.1x10^6^ ± 7.1x10^5^ for *P. aeruginosa*, and 4.1x10^7^ ±6.1x10^7^ for *S. aureus*. Stainless steel control (SS). % Reduction was calculated according to the EPA protocol (EPA 2008) and % Recovery was calculated as the ratio mean (RLU/20cm^2^) surface products to mean (RLU/20cm^2^) SS.
